# A ciliated adenocarcinoma of the lung mimicking lung abscess and pneumonia

**DOI:** 10.1002/rcr2.1407

**Published:** 2024-06-10

**Authors:** Naoki Fujimoto, Kohei Fujita, Ayami Ishida, Koki Moriyoshi, Kiminobu Tanizawa

**Affiliations:** ^1^ Division of Respiratory Medicine, Center for Respiratory Diseases National Hospital Organization Kyoto Medical Center Kyoto Japan; ^2^ Department of Diagnostic Pathology National Hospital Organization Kyoto Medical Center Kyoto Japan

**Keywords:** ciliated adenocarcinoma, lung abscess, lung adenocarcinoma, non‐small cell lung cancer, pneumonia

## Abstract

This case report describes a 78‐year‐old man initially treated for pneumonia and lung abscess who was resistant to antimicrobial treatment and was eventually diagnosed with ciliated adenocarcinoma. Ciliated adenocarcinoma, a rare non‐terminal respiratory unit (TRU)‐type lung adenocarcinoma, presents a unique diagnostic challenge because of its similarity to pneumonia and lung abscesses. Morphologically, the ciliated adenocarcinoma in this case appeared to be a non‐TRU type adenocarcinoma, with partial mucous epithelium, no visible extracellular mucus, thyroid transcription factor (TTF)‐1 negativity, and mucin (MUC) 5AC positivity on immunostaining. The patient was considered to have ciliated adenocarcinoma based on the fact that the mucous epithelium was partial and extracellular mucus was not prominent. This case emphasizes the importance of considering malignancy in patients with non‐resolving pulmonary infections.

## Graphical abstract

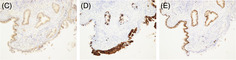

## INTRODUCTION

Ciliated adenocarcinoma is a rare type of lung adenocarcinoma that is not described in the World Health Organization (WHO) classification system. Lung adenocarcinoma can be histologically classified into terminal respiratory unit (TRU)‐type adenocarcinoma and non‐TRU type adenocarcinoma; ciliated adenocarcinoma belongs to the latter type [1]. Non‐TRU adenocarcinomas originate from the central bronchial epithelium and do not express TTF‐1. These tumours are typically characterized by dense tissue, poor morphological differentiation, and frequent necrosis [2], making them difficult to distinguish from malignant findings. On radiological imaging, the lesions are invasive and cavitary and, at first glance, appear to be pneumonia or lung abscess, making it difficult to distinguish them from malignant findings. Here, we report a case of ciliated adenocarcinoma diagnosed by bronchoscopy after initial medical treatment for pneumonia and a lung abscess resistant to antimicrobial treatment.

## CASE REPORT

**FIGURE 1:**
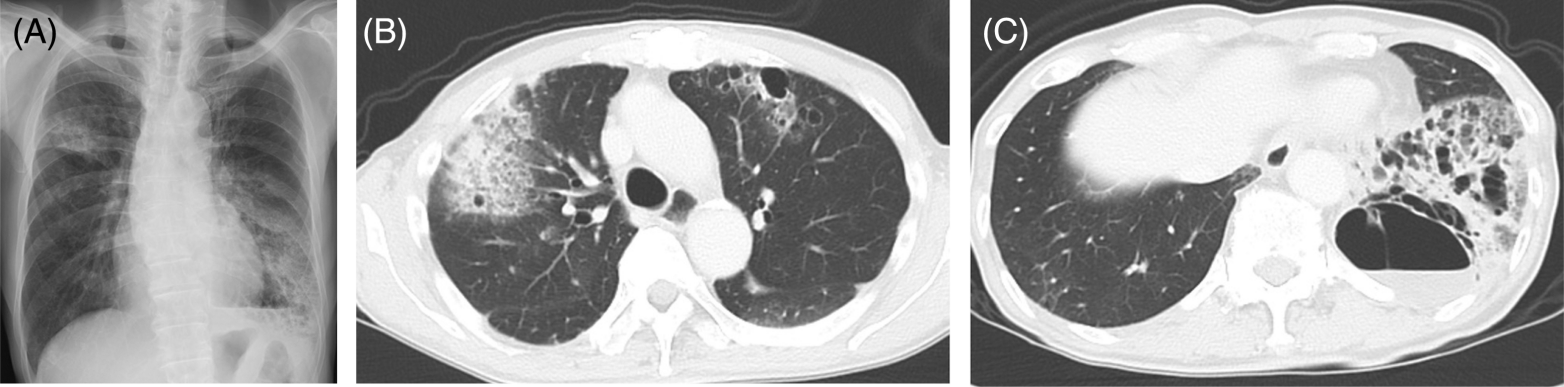
Chest x‐ray at initial examination (A). Chest computed tomography scan showing infiltrating shadow and bronchiectasis in the right upper lobe (B) and infiltrating shadow and cavitary lesions in the left lower lobe (C).

**FIGURE 2:**
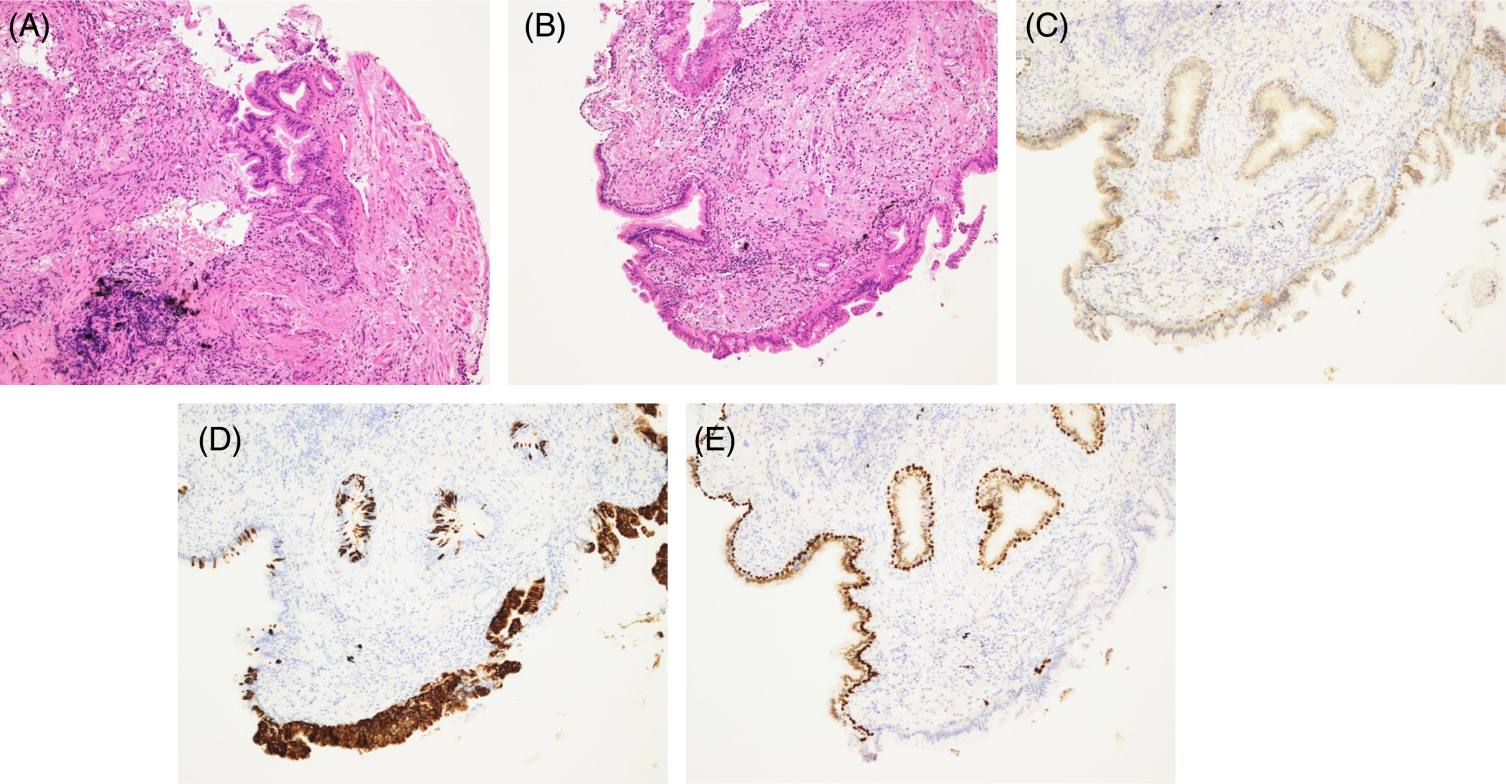
Pathological images of haematoxylin and eosin staining of the tissue obtained from the right upper lobe (A) and the left lower lobe (B). The two tissues have the same morphology, strongly suggesting that they are the same tumour. Pathological images of the left lower lobe showing thyroid transcription factor (TTF)‐1 negativity (C) and mucin (MUC)5 AC positivity (D) on immunostaining and no bilayer on p63 staining (E), consistent with the features of ciliated adenocarcinoma.

A 78‐year‐old man presented to his family physician with a nocturnal cough that had persisted for over a month. He was referred to our hospital after chest radiography revealed abnormal shadows in the right upper and left lower lung fields suggestive of pneumonia (Figure 1A). A chest computed tomography (CT) scan showed infiltration, ground‐glass opacities and bronchiectasis in the right upper and left lower lobes, multiple cavitary lesions scattered throughout both lungs, and an abscess cavity in the left lower lobe (Figure 1B,C). He was admitted to the hospital with suspected pneumonia and lung abscess. He had a history of smoking; however, his medical history included only hypertension. Upon admission, the patient had no fever, dyspnoea, or chest pain, and his main symptom was a productive cough. Blood tests showed a C‐reactive protein level of 0.79 mg/dL and a white blood cell count of 9.2 × 10^3^/μL, and tumour markers such as carcinoembryonic antigen and carbohydrate antigen 19–9 were only slightly elevated at 4.90 ng/mL and 106 U/mL, respectively. Immediately after admission, CT‐guided drainage of the abscess cavity in the left lower lobe was performed for the purpose of the identification of the causative pathogenic microorganisms and controlling the source of infection, and sulbactam and ampicillin were initiated as treatment for the lung abscess. *Haemophilus influenzae* was detected in the aspirated pus, prompting a switch to ceftriaxone, and antimicrobial therapy was continued for 1 week. However, there was no improvement in the clinical symptoms or imaging findings compared to those at the time of admission. Considering the possibility of malignancy, a bronchoscopy was performed for further evaluation. Bronchoscopic biopsies were obtained from the lower left and upper right lung lobes. The pathology of both biopsy tissues revealed ciliated adenocarcinoma, a rare form of adenocarcinoma that is completely identical (Figure 2A–E). Following a comprehensive evaluation, the clinical stage was diagnosed as cT4N1M1a stage IVA due to the presence of ciliated adenocarcinoma in the contralateral lung. The Oncomine Dx Target Test revealed no actionable driver oncogene alterations. Additionally, programmed death‐ligand 1 (PD‐L1) expression assessed by Dako 22C3 assay showed that PD‐L1 expression was less than 1%. Currently, the patient is undergoing combined immunotherapy.

## DISCUSSION

This case illustrates the complexity in diagnosing lung adenocarcinomas, which are the most prevalent type of lung cancer, constituting 40% of all non‐small cell lung cancer cases. From a histological perspective, these can be categorized as TRU‐ and non‐TRU‐type adenocarcinomas. TRU‐type adenocarcinoma originates from peripheral type II lung cells or Clara cells and exhibits positivity for thyroid transcription factor (TTF)‐1 [1]. In contrast, non‐TRU‐type adenocarcinomas originate from the central bronchial epithelium, which is devoid of TTF‐1 expression. Non‐TRU‐type adenocarcinomas are typically characterized by a firm texture, poor morphological differentiation, and frequent necrosis [2]. Moreover, they have been reported to express mucin (MUC) 5AC and MUC5B, as evidenced by immunostaining [3]. It has been proposed that ciliated adenocarcinomas evolve through mucinous columnar cell transformation and progress to dysplasia. Despite being classified as a non‐TRU‐type adenocarcinoma, ciliated adenocarcinomas exhibit distinct clinical, histological, immunohistochemical, and molecular biological features [1]. In the current case, the carcinoma was morphologically considered an adenocarcinoma of the non‐TRU type. However, the mucous epithelium was only partially present and the extracellular mucus was inconspicuous, which is typical of invasive mucinous adenocarcinoma. Immunostaining was negative for TTF‐1 and positive for MUC5AC, suggesting ciliated adenocarcinoma. Ciliated adenocarcinoma is a diagnosis that has not yet been established according to the WHO classification, and further research is warranted. [1] In addition, the characteristics of non‐TRU adenocarcinomas are still not fully understood, although they have been reported to correlate with MUC5AC expression, MUC5B expression, and KRAS mutations and to have a poorer prognosis than TRU adenocarcinomas [4]. Further studies are required to characterize non‐TRU adenocarcinomas, which may be useful for selecting appropriate patients for specific therapies.

Additionally, this patient initially presented with characteristics of pneumonia and lung abscess, such as infiltrative shadows and cavitary lesions. Fluid retention in cysts rarely responds to antimicrobial therapy alone. Clinical success rates with combined appropriate antimicrobial therapy and effective drainage can reach 75%–90% [5]. Therefore, in this case, early CT‐guided drainage was employed. In addition, *H. influenzae* was detected in the CT‐guided abscess puncture fluid and cytology revealed no evidence of malignancy, which made pneumonia and lung abscesses more strongly suspected, and treatment was initiated with a focus on these conditions. Therefore, there was a delay of approximately 10 days in pathological diagnosis. Distinguishing between malignant and benign diseases is often challenging when cavitary lesions are present in the lungs. Several cases of lung abscesses have been reported in which malignancy was initially considered as a possible diagnosis with treatment that failed to respond to appropriate antibiotics with or without percutaneous drainage [6]. Since the incidence of infection is increased in patients with malignancy and the lungs are the preferred site of infection, it is imperative to consider the coexistence of infection and malignancy in the thoracic cavity. When lung abscesses do not improve with appropriate medical therapy and lung cancer is suspected, invasive diagnostic procedures should be aggressively pursued.

## AUTHOR CONTRIBUTIONS

N.F. drafted the manuscript. K.F. and K.T. revised the manuscript. N.F., K.F., and K.T. treated this patient. A.I. and K.M. evaluated and interpreted the pathologies. All authors have revised and reviewed the manuscript for intellectual content. All the authors approved the final version of the manuscript.

## CONFLICT OF INTEREST STATEMENT

None declared.

## ETHICS STATEMENT

The authors declare that they have obtained appropriate written informed consent from this patient for the publication of this manuscript and accompanying images.

## Data Availability

Data available on request from the authors.
